# Dominance of the ON1 Genotype of RSV-A and BA9 Genotype of RSV-B in Respiratory Cases from Jeddah, Saudi Arabia

**DOI:** 10.3390/genes11111323

**Published:** 2020-11-09

**Authors:** Hessa A. Al-Sharif, Sherif A. El-Kafrawy, Jehad M. Yousef, Taha A. Kumosani, Mohammad A. Kamal, Norah A. Khathlan, Reham M. Kaki, Abeer A. Alnajjar, Esam I. Azhar

**Affiliations:** 1 Biochemistry Department, Faculty of Sciences, King Abdulaziz University, Jeddah 21589, Saudi Arabia; halsharif0050@stu.kau.edu.sa (H.A.A.-S.); tkumosani@kau.edu.sa (T.A.K.); 2 Special Infectious Agents Unit, King Fahd Medical Research Center, King Abdulaziz University, Jeddah 21589, Saudi Arabia; saelkfrawy@kau.edu.sa (S.A.E.-K.); nalkhathlan@kau.edu.sa (N.A.K.); rmkaki@kau.edu.sa (R.M.K.); abeeralnajjar@yahoo.com (A.A.A.); 3 Department of Medical Laboratory Sciences, Faculty of Applied Medical Sciences, King Abdulaziz University, Jeddah 21589, Saudi Arabia; 4 Biochemistry Department, College of Sciences, Jeddah University, Jeddah 21589, Saudi Arabia; jyousef@kau.edu.sa; 5 King Fahd Medical Research Center, King Abdulaziz University, P. O. Box 80216, Jeddah 21589, Saudi Arabia; prof.ma.kamal@gmail.com; 6 Enzymoics, Novel Global Community Educational Foundation, 7 Peterlee Place, Hebersham, NSW 2770, Australia; 7 Pediatric Department, Faculty of Medicine and Pediatric Intensive Care Unit, King Abdulaziz University Hospital, King Abdulaziz University, Jeddah 21589, Saudi Arabia; 8 Department of Medicine, Department of Infection Disease, and Department of Infection Control and Environmental Health, King Abdulaziz University Hospital, King Abdulaziz University, Jeddah 21589, Saudi Arabia

**Keywords:** RSV-A, RSV-B, phylogenetic analysis, Saudi Arabia

## Abstract

Human respiratory syncytial virus (HRSV) is a main cause of hospital admission for lower respiratory tract infection. In previous studies from Saudi Arabia, higher prevalence of the NA1 genotype in group A was observed from Riyadh and Taif. This study recruited respiratory cases from Jeddah during January to December, 2017. RSV represented 13.4% in the recruited cases with 64% of them belonging to group A and 36% to group B. All group A cases in this study were ON1 type characterized by duplication of 72 nucleotides, 24 amino acids in the C-terminal in the second hypervariable region of the G gene. In addition, for group B all of the cases were clustered under BA9, which had uniquely characterized as duplication of 60 nucleotides in the G protein. Our sequences showed similarity with earlier sequences from Saudi Arabia, Kuwait, Thailand, South Africa, Spain, the USA and Cyprus. Some amino acid substitutions in the investigated sequences would cause a change in potential O-glycosylation and N-glycosylation profiles from prototype ON1. The predominance of the ON1 and BA9 genotype of RSV-A in Jeddah compared to previous Saudi studies showing predominance of the NA1 genotype for group A. This difference in genotype prevalence could be due to fast spread of the ON1 genotype worldwide or due to the flux of travelers through Jeddah during hajj/umrah compared to Riyadh and Taif. This shift in genotype distribution requires continuous surveillance for genetic characterization of circulating respiratory infections including RSV. These findings may contribute to the understanding of RSV evolution and to the potential development of a vaccine against RSV.

## 1. Introduction

Human respiratory syncytial virus (HRSV) is the most common viral infection that induces acute lower respiratory tract infections (ALRTIs) in children, elderly people and immune-suppressed patients [1,2,3]. It is also one of the main causes of hospital admission due to severe acute lower respiratory infection (ALRI) [3]. The recurrences of HRSV infection are known throughout life, particularly in children less than 5 years of age [2]. It causes mild to severe illness such as pneumonia and bronchiolitis [1]. A systematic review and meta-analysis [3] for RSV studies in young children from January 1995 to June 2009 estimated that 33.8 million cases of RSV associated with ALRI occurred worldwide in children younger than 5 years (22% of ALRI cases), with 3.4 million who had severe RSV-associated ALRI needing hospital admission in 2005. They also estimated 66,000–199,000 RSV-associated deaths among children less than 5 years old. They concluded that the incidence and mortality can vary substantially from year to year in any one setting [3].

Human RSV belongs to the *Pneumovirus* genus of the *Paramyxoviridae* family, now the genus *Orthopneumovirus* in the *Pneumoviridae* family. It is an enveloped, non-segmented virus with negative-sense, single-stranded RNA. The genome size is around 15.2 kb and encodes for 11 proteins: NS1, NS2, N, P, M, SH, G, F, M2-1, M2-2, and L [2]. The attachment glycoprotein (G protein) is the major variable region used to study the genetic diversity of HRSV within and between their genotypes. The hypervariable regions located in ectodomain at the C-terminal end of G protein [1]. Based on genetic variations in G protein, HRSV is divided into two groups: group A which is categorized into 14 genotypes (GA1 to GA7, SAA1, CB-A, NA1 to NA4 and ON1) and group B which is subdivided into a further 27 genotypes (BA1 to BA12, BA-C, SAB1 to SAB4, GB1 to GB4, URU1 to URU2, CB-B, CB-1, BA-CCB and BA-CCA) [2,4]. The subtype ON1 of RSV-A was first identified in Canada in December of 2010 and was reported to contain a unique duplication of 72 nucleotides in the second hypervariable region of the G gene [5] and it may be spreading rapidly and replacing the NA1 subtype in many countries such as Saudi Arabia, South Korea, Italy, Germany, Bulgaria, Croatia and China [6,7,8,9,10,11,12,13]. A similar duplication was reported in the variable region around 60 nucleotide of the G protein of the BA subtype of RSV-B in 1999 in Argentina [14].

The lack of available vaccines increases the circulation of RSVs every season, which induce mild to serious disease in children and elderly adults. Although the virus was first isolated in 1956, there are currently no safe and effective vaccines licensed for human use. The development of novel prevention and treatment strategies should be considered a priority [3]. The G gene is under constant pressure from RSV-specific neutralizing antibodies because it induces a protective immune response. This has made the G gene of RSV a part of several candidate vaccines [15].

In Saudi Arabia, a study from Riyadh with a sample collected in 2007–2008 showed that about 59% of the positive RSV cases was of group A that clustered within the NA1 genotype where 41% of samples were group B under the BA clade [16]. Another study also from Riyadh in 2014 [6] showed the co-circulation of RSV genotypes in children less than 5 years of age. Phylogenic analysis showed that 77% of the subjects clustered with group ARSV in the NA1 (80.95%) and ON1 (19.05%) genotypes and 23% of the subjects clustered with group B viruses in the BA9 genotype with duplication in the hypervariable region in the G gene in the ON1 group and BA group.

This study adds more information to the limited data on the diversity and typing of the HRSV in Saudi Arabia. Better understanding of the extent, diversity and evolution of HRSV in Jeddah, Saudi Arabia, will give more insight into the molecular characters within a global context. The importance of this study comes from the reporting of strains circulating in Jeddah due to its geographical location as a travel hub connecting millions of pilgrims who come to visit the holy places in Makkah and Al-Madina all year round. The travel of pilgrims in and out of Jeddah plays a major role in the spread of infectious diseases inside the city and hence the region, as well as to the home countries of the pilgrims. The data generated from this study is expected to provide better understanding of the virus evolution and movement in and out of the country.

## 2. Materials and Methods

### 2.1. Inclusion and Exclusion Criteria

The recruited subjects included respiratory infection patients hospitalized at the intensive care unit of King Abdulaziz University Hospital between January and December of 2017 without discrimination of age and gender. The only exclusion criteria was the refusal of the patient to participate in the study.

### 2.2. Sample Collection

Samples were sent to the Special Infectious Agents Unit for routine diagnosis of the respiratory infectious agents. Ethical approval was obtained from the Unit of Biomedical Ethics in King Abdulaziz University hospital (approval number 290-17). Samples included upper and lower respiratory tract specimens (nasopharyngeal swab (NP), throat swab (TS) and sputum (S)).

### 2.3. Molecular Detection of Respiratory Viruses

Total nucleic acids (NA) were extracted from clinical samples using a MagNaPure Compact Nucleic Acid Isolation Kit I (Roche, Germany) in which 400 μL of respiratory specimens were used and eluted in 100 μL according to the manufacturer’s instructions. The detection of respiratory syncytial virus (RSV) and other respiratory pathogens was routinely performed using a Fast-Track Diagnostics^®^ Respiratory 33 Kit (Fast-track Diagnostics, Luxembourg), according to the manufacturer’s instructions, on Applied Biosystems^®^ 7500 Fast (Applied Biosystems, Foster City, CA, USA). All positive samples were kept at −80 °C for further molecular analysis.

### 2.4. Virus Isolation

RSV positive clinical specimens (100 µL) were filtered and inoculated on human cervix epithelial adenocarcinoma HeLa cells seeded in 24-well plates in 1.5 mL of Dulbecco’s modified Eagle’s medium (DMEM) supplemented with 10% fetal bovine serum (FBS) (Gibco, Gaithersburg, MD, USA). The samples were incubated with the HeLa cells for 1.5 h with rocking every 15 min. Next, 1.5 mL of maintenance medium was added to each well and incubated at 37 °C, 5% CO_2_. Cytopathic effect (CPE) was observed daily for 10 days and then cells were scraped and medium with cells (virus-cell suspensions) were collected, aliquoted and stored at −80 °C. The isolated virus was used for whole genome sequencing of RSV-A.

### 2.5. Whole Genome Sequencing and Typing of RSV

For RSV typing, the extracted viral RNA from positive RSV samples were reverse transcribed into cDNA, using SuperScript™ III Reverse Transcriptase (Invitrogen, Carlsbad, CA, USA) using 10 µL of total RNA with 1 µL of 2 µM deoxyribonucleotide triphosphates (dNTPs), 1 µL nuclease free water and 1 µL of 10 µM random dodecamer (12N), heated at 65 °C for 5 min on a Mastercycler^®^ Pro S (Eppendorf, Ocala, FL, USA) and quickly chilled on ice for at least 5 min. Next, 4 µL of First-Strand Buffer (5×), 1 µL of 0.1 M dithiothreitol (DTT), 1 µL of 40 U/µL RNasin^®^ PLus RNase Inhibitor (Promega, Madison, WI, USA) and 1 µL of 200 U/µL SuperScript III Reverse Transcriptase were added to the mixture, followed by incubation at 25 °C for 5 min, 50 °C for 60 min and 70 °C for 15 min.

RSV subgroup A and B typing were performed by amplifying the G gene with subgroup-specific primers according to Xia et al. for type A [17] and Runan et al. for type B [18]. The amplicon products were 1320 bp and 868 bp for subgroups A and B, respectively. The bands with the correct size were excised from the agarose gel and purified using a QIAquick^®^ Gel Extraction Kit (Qiagen, Hilden, Germany) following the manufacturer’s protocol. The full length of the RSV-A genome was covered by sequencing 13 overlapping PCR fragments according to Rebuffo et al. [19].

All PCR products, either for RSV-A typing or whole genome sequencing, were sequenced using an ABI Prism Big Dye Terminator Cycle Sequencing Kit v3.1 on an ABI 3500 Genetic Analyzer (Applied Biosystems, Foster City, CA, USA) in both directions. The cycle sequence product was purified using Sephadex^®^ with 0. 45 um MultiScreen-HV.

Genome assembly and phylogenetic analysis was performed using MEGA 7 (v.7, Pennsylvania State University, University Park, PA, USA) and Geneious R09 software [20]. The prediction of potential N-glycosylation (Asn-X-Ser/Thr, where X is any amino acid (AA) except proline) and O-glycosylation sites (Ser or Thr) in G-protein was performed using NetNGlyc 1.0 [21] and NetOGlyc 3.1 [22]. The sequences of deduced amino acid in the G protein of RSV-A group were compared to the prototype strain of RSV-A ON1 genotype from Canada (GenBank accession number JN257694) wherein the G protein of the RSV-B group were compared to the prototype strain of BA genotype from Argentina (GenBank accession number AY333364).

## 3. Results

### 3.1. The Characterization and Prevalence of RSV in Respiratory Samples

A total of 834 clinical samples were collected between January and December 2017. Samples were routinely tested using the FTD 33 respiratory pathogens kit. Respiratory pathogens were identified in 74% of the total number of samples. Among them 84 (13.4%) were positive for RSV. The mean age for RSV patients was 8.33 years ±17.9 (<1 month–69 years). The majority of RSV-infected cases (71.4%) were under 1 years old, as seen in Table 1, with males representing 54.8% of patient. Among the positive RSV cases, 59.6% were admitted to the emergency room then released without hospitalization, and 68.4% of cases were younger than 1 years old (Table 1).

**Figure 1:**
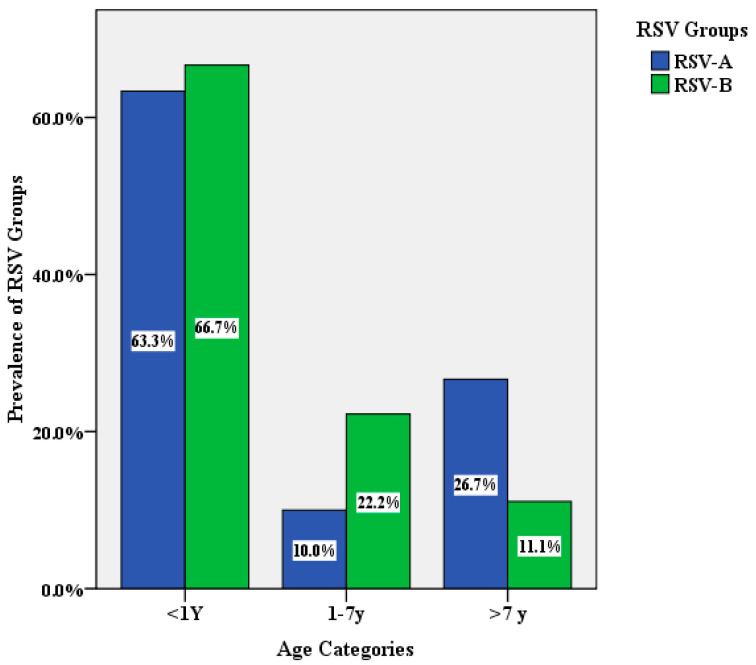
The age distribution of human respiratory syncytial virus (HRSV) groups in the recruited cases.

Amplification of the G gene was successful in 50 of the RSV positive samples using RSV-A type-specific primers targeting the G gene. The resulting PCR products were subjected to DNA sequencing. Type-specific PCR identified group A RSV in 32 samples (64%) and group B RSV in 18 (36%) of the RSV positive samples. (Figure 1). The generated sequences were submitted to the GenBank database and were given the accession numbers MN434097-MN434127 for RSV-A and MW019650-MW019667 for RSV-B.

### 3.2. Phylogenetic Analysis of RSV

**Figure 2:**
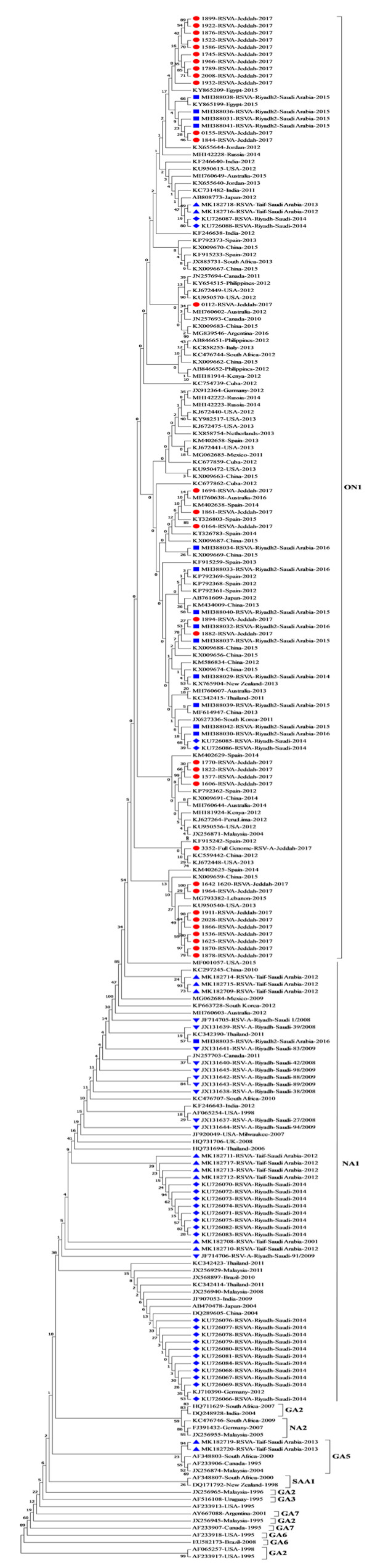
Phylogenetic analysis of G-gene of RSV group A in Jeddah, Saudi Arabia (2017). Saudi sequences in this study are labeled with a red square and reference strains are labeled as accession number-country-year of isolation; Saudi reference strains isolated in 2014 are labeled with a blue diamond and those isolated in 2014–2016 are labeled with a blue circle; the Riyadh samples isolated in 2008–2009 are labeled with an inverted triangle and Taif samples are labelled with a blue triangle Phylogenetic trees were constructed using the neighbor-joining method after being aligned with CLUSTALW using MEGA 7.

Phylogenetic analysis of the generated sequences for the 31 RSV-A positive samples with other reference sequences from GenBank showed that all samples clustered with the ON1 genotype. This genotype is characterized by the insertion and duplication of 72 nucleotides and 24 amino acids, in the C-terminal end in the second hypervariable region of the G gene (Figure 2). The duplication starts after nucleotide 847 in the G gene (position numbers refer to the RSV-A isolate, accession no. HRU39662). This duplication in the G gene was found in 27 samples. The overall average of pairwise distance of variation in the G gene between the reported Saudi samples and the ON1 prototype reference was 0.073.

**Figure 3:**
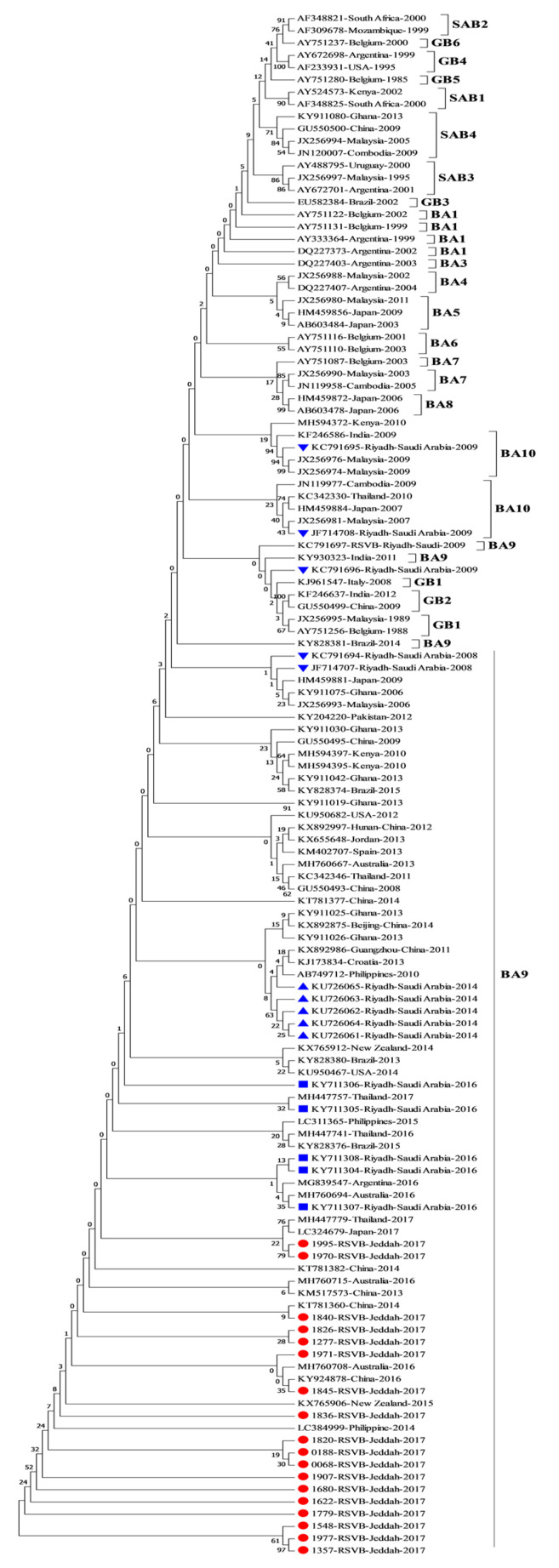
Phylogenetic analysis of the G gene of RSV group B in Jeddah, Saudi Arabia (2017). Saudi sequences in this study are labeled with a red square and reference strains are labeled as accession number-country-year of isolation; Saudi reference strains isolated in 2014 are labeled with a blue diamond and those isolated in 2014–2016 are labeled with a blue circle; the Riyadh samples isolated in 2008–2009 are labeled with an inverted triangle and Taif samples are labelled with a blue triangle. Phylogenetic trees were constructed using the neighbor-joining method after being aligned with CLUSTALW using MEGA 7.

The results of phylogenetic analysis for the 18 RSV-B samples showed that they belong to the BA genotype, specifically the BA9 genotype (Figure 3). All of the sequences had insertion of 60 nucleotides after position 719, and a deletion of CCAAAA was also observed at the position 475 (P159 & K160) in all RSV-B samples (nucleotides position numbers refer to the RSV-B position, accession no. AY333364). The overall average of pairwise distance of variation in the G gene between all of RSV-B samples and other references including the prototype was 0.09.

### 3.3. Amino Acid Sequence Analysis of the G Gene

**Figure 4:**
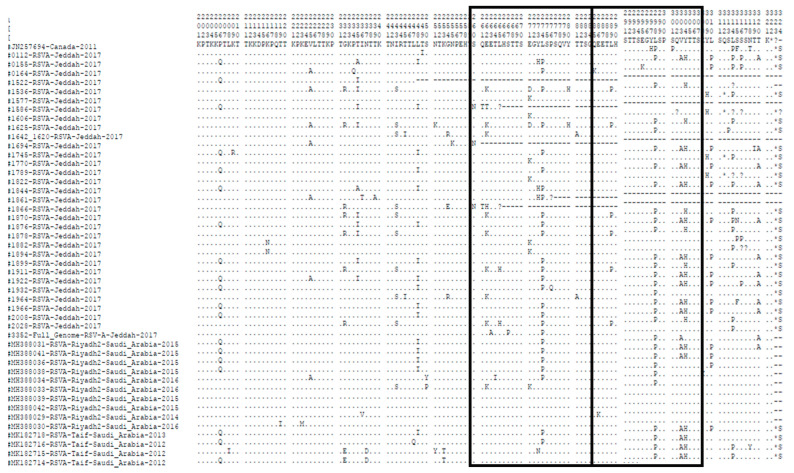
The sequences alignment of deduced amino acid in the second hypervariable region of the G protein of he RSV-A group for the ON1 strain are shown to correspond to the prototype strain of ON1 from Canada (GenBank accession number JN257694). The positions of the amino acids correspond to 213–320 of the G protein. Dots indicate identical residues, and asterisks indicate the position of stop codons. Boxes frame the 24 amino acid duplicated region.

The length of the G gene protein in most of the investigated ON1 strains in this study was 322aa with a TGA as a stop codon similar to the prototype ON1 strain from Canada. Four samples had a different stop codon (TAA) and were 312 amino acids long. One sample had a missing amino acid (L) at position 314 and another sample was missing two amino acids (SS) at position 316 and 317, so their lengths were 321 and 320, respectively. Comparing ON1 amino acid sequences in this study with the prototype ON1 strain in the duplicated region, we observed amino acid changes in the following positions: L298P in 19, L274P in 18 samples, Y304H in 16 samples, L310P in 11 samples, V303A in 10 samples, E271D\K in 8 samples and E262K in 7 samples. The nucleotide and amino acid sequences variability between our sequences and the ON1 prototype are shown in Table 2 and Figure 4.

**Figure 5:**
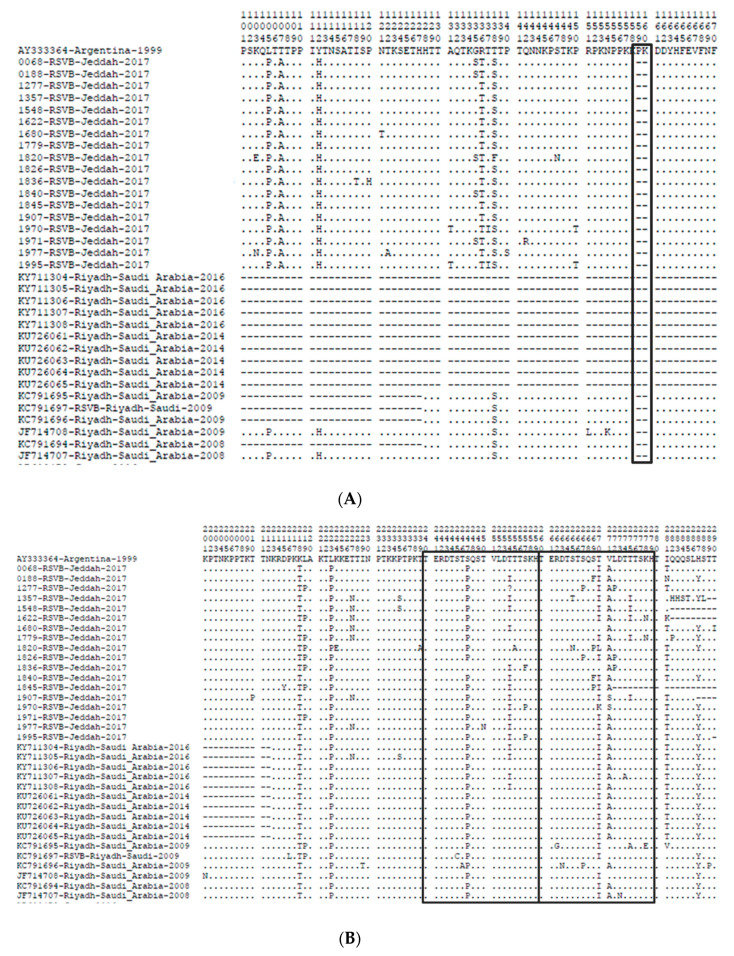
The sequences alignment of deduced amino acid. (**A**) The deletion part in our samples. (**B**) The deduced amino acid in the second hypervariable region of the G protein of the RSV-B group for the BA strain shown to correspond to the BA prototype strain from Argentina (GenBank accession number AY333364). The positions of amino acids correspond to 213–320 of the G protein. Dots are indicated for identical residues, and asterisks indicate the position of stop codons. Boxes frame the 20 amino acid duplicated region.

In addition, the BA strains in RSV group B in this study showed variations in the protein length of the G gene due to the site of the stop codon. The G gene of three samples of this group had a TAA as a stop codon with a length of 313 amino acids, which is 3 amino acids shorter than the prototype BA strain from Argentina (316 amino acids), and one sample had a G gene 292 amino acids long with a TAG stop codon as in the prototype. Two samples had a TAG stop codon and a longer protein (319 or 320 amino acids) than the prototype due to the addition of three or four amino acids. We were not able to obtain a full-length G gene for 11 samples, which were missing between 12 and 34 amino acids at the end of the gene. More than 30 mutations in the sequences were identified in the G gene when compared to the prototype strain. All RSV-B samples had the same three substitutions, K218T, L223P and S247P, except one sample that did not have the mutation at position 247 in the duplicated region. All samples had a mutation at position 270, 14 samples had the substitution T270I, while other mutations (T270L, T270K and T270F) where detected individually in each different sample. There was also an amino acid change for the valine at the position 248 in 17 samples. I281T substitution was detected in 16 BA strains in this study while I281N and I281K were detected in one sample each. At site 287, most of the samples (ten samples) had H287Y substitutions. The nucleotide and amino acid sequences variability between our sequences and the BA prototype are shown in Table 3 and Figure 5.

### 3.4. N- and O-Glycosylation Site Analysis in Amino Acid Sequence

Analyzing the N-glycosylation sites showed three to eight sites in the investigated RSV-A sequences. Only one of them (at aa 85, NTT) was conserved among all the sequenced samples and was reported in the prototype ON1. The N-glycosylation sites at aa 103(NLS), 135(NTT) and 237(NTT) were found in all except three sequences. The change of amino acid threonine at position 320 into alanine (T320A) caused the loss of a potential N-glycosylation site in 10 samples. There were three unique substitutions that led to the gain of possible N-glycosylation sites (Q127N, T198N, S315N) in three different samples, whereas for the investigated RSV-B sequences, two to four N-glycosylation sites were predicted in the sequences of the samples. Two of them (at AA 81 and 86, NHT and NIT) reported in the BA prototype were conserved among all samples. At residues 296 and 310 (NST) were found in seven and five of our sample sequences, respectively. The amino acid substitution S/N at position 265 caused a gain of potential N-glycosylation site in one sample (NTS). Two samples had substitutions (K278N) that led to a gain of possible N-glycosylation sites (NHT).

The number of O-glycosylation sites in the investigated RSV-A sequences varies from 52 to 86. All the 10 potential O-glycosylation sites reported in the prototype ON1 strain in the duplication region (threonine at 264, 268, 269, 281 and 282 and serine at 267, 270, 275, 277 and 283) were also found in the strains from this study except for three different samples due to substitution at S267P in one sample and T281A in two samples. Two samples showed amino acid substitution Q261T, which was not found in the prototype ON1 strain and gave rise to an additional potential O-glycosylation site, whereas for RSV-B, the number of O-glycosylation sites varies from 27 to 34. Before the duplication region there were three residues common with the BA prototype at site numbers 228, 232 and 236. In the duplication region, there were two sites at 260 and 266.

### 3.5. Full Genome Sequence

**Figure 6:**
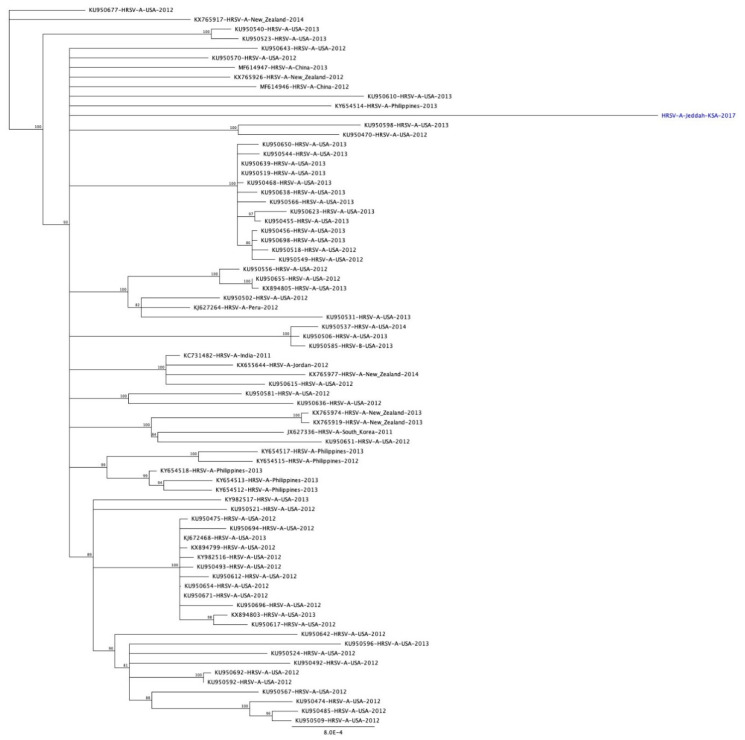
Phylogenetic analysis of full genome RSV group A in Jeddah, Saudi Arabia (2017). Saudi sequences in this study are labeled with (●) and reference strains are named as accession number-country-year of isolation. A phylogenetic tree was constructed the using neighbor-joining method after being aligned with the CLUSTALW using MEGA 7.

In this study, the whole genome of RSV-A from a clinical specimen was sequenced. The full genome was 15,225 bp in length including a 72 nucleotides duplication in the C-terminus of the G gene. From these, 11 open reading frames encoded viral proteins for the NS1, NS2, N, P, M, SH, G, F, M2-1, M2-2 and L genes. The similarity of amino acid sequences with other reference sequences from GenBank (Figure 6) ranged from 99.032% to 99.220%, and the GC content was 33.4%.

## 4. Discussions

Respiratory syncytial virus (RSV) is the most common pathogen causing LRTIs among children and older adults in Saudi Arabia and worldwide [23,24,25]. RSV was reported to have the highest infection rate (97.4%) among the total viral agents in Saudi patients under 2 years of age with associated signs and symptoms of ARTIs between January 2013 and December 2014 [26]. A large study from South Africa (with 13,664 patients) between February 2009 to May 2012 showed that RSV was detected in 16% of the recruited subjects with more than 50% of them below 4 years of age [27]. G protein in the RSV is responsible for the virus attachment to the ciliated cells of the host airways and mediates virus adherence [28]. Therefore, G protein is the target for neutralizing antibodies but because of its high genetic variability and higher numbers of glycosylation sites it gives the virus the ability to evade the immune system and delay antigen recognition [29].

In this study, we report the genetic diversity of the G gene of RSV-A and B as well as the first full genome sequence of RSV-A from Jeddah, Saudi Arabia. We also compared the generated sequences with other sequences in GenBank to identify the genetic relatedness of the local isolates to the other reported isolates worldwide.

RSV was detected in 13.4% of the recruited positive respiratory cases using real-time RT-PCR; 71.4% of the RSV positive cases were children less than one year old, which is in accordance with surveillance studies performed in other parts of the world [30]. Group RSV-A was predominant with 64% among positive RSV cases. Similar predominance of RSV-A over RSV-B was reported earlier in Saudi Arabia, Germany, Thailand, South Africa, Spain, USA, France, Cyprus and Kuwait [6,8,31,32,33,34,35,36,37].

Phylogenetic analysis (Figure 2) showed that all of the RSV-A samples enrolled in this study clustered with strains of the ON1 genotype, which contains the largest duplication in the G gene, first reported in 2010 in Ontario, Canada [5], which is in accordance with other recent studies (2017–2019) from Lebanon, Iran, France, Vietnam, Egypt, Mexico and Spain that showed the predominance of the ON1 genotype, indicating the rapid spread of the ON1 strain in different parts of the world [25,30,33,35,38,39,40]. Two studies performed in Riyadh, Saudi Arabia, showed the predominance of the NA1 strain of RSV-A in 2008 and 2012. One study performed in 2008 showed that the NA1 strain was the predominant genotype (90.9%) in the Riyadh region with no incidence of the ON1 strain [41], while the other study [6], also performed in Riyadh, in 2012, showed the first incidence of the ON1 genotype, but the NA1 genotype was still the predominant genotype (82.6%). Saudi strains from Taif (2019), reported by Al Aboud et al. [42], for samples collected in 2012, belong to the NA1 genotype (6/13, 46.15%), ON1 (5/13; 38.46%) and GA5 genotype (2/13; 15.38%). Recent sequences retrieved from GenBank (accession numbers MH388029 to MH388042) for samples collected from Riyadh, Saudi Arabia, between 2014 and 2016, showed 14 RSV-A samples of the ON1 type, but the work was not published, and we have no prevalence data on this study.

Collectively, these data show the rapid emergence of ON1 in Saudi Arabia from 0% in 2008 to 17.4% (Riyadh) and 38.46% (Taif) in 2012 and an uncertain dominance in 2014–2016 (sequence data only) and the predominance of ON1 in this study. The predominance of the ON1 genotype in this study compared to the previous studies conducted in Saudi Arabia could be either due to the rapid spread of the ON1 genotype over the years from its first identification in 2010 and spread all over the world or due to the difference in the sample collection time between our study and the previous studies. The third possible explanation is the different sample collection sites (Riyadh and Taif vs. Jeddah) as Jeddah is a travel hub for pilgrims coming from all over the world to visit the Holy places in Makkah and Madinah, which makes the city more susceptible to exposure to more diverse infectious agents, including respiratory infections, with the flux of pilgrims entering through the city.

Phylogenic analysis showed that the sequences of the investigated samples in this study clustered with several lineages of the ON1 clusters (Figure 2). Nine of the samples clustered with sequences from Saudi Arabia isolated in 2016, Lebanon (2015) and the USA (2013) and were close to isolates from Italy (2013) and Philippines (2012). Ten samples grouped with isolates from Saudi Arabia (2015), China (2015), the Philippines (2012), Spain (2012), the USA (2013), Jordan (2012), Russia (2014) and Kenya (2012). Five samples clustered with isolates from Austria (2016) and Spain (2014, 2015). Four samples grouped with isolates from Saudi Arabia (2015), Thailand (2011), the USA (2012, 2013), China (2012, 2015), Russia (2014), Spain (2012, 2014) and the Netherlands (2013). The sequences of two samples grouped with isolates from Saudi Arabia (2016), Spain (2012, 2013), Egypt (2015), Kenya (2012), Argentina (2016), China (2014, 2015), Austria (2013), South Africa (2013), Cuba (2012), Peru (2012) and the USA (2012, 2013). One sample grouped with isolates from Germany (2012), Saudi Arabia (2015), China (2013), the USA (2015), Jordan (2013), Argentina (2016) and Kenya (2014). Another sample grouped with isolates from South Korea (2011), Spain (2013, 2014), Saudi (2014), Kenya (2012), South Africa (2012), Argentina (2016) and China (2012) [6,9,25,32,33,34,38,42,43,44,45,46,47,48,49,50,51].

The full genome sequence for the RSV1-A isolate from this study had a genome of 15,225 nucleotides long. It showed similarity to prototype ON1, except for six amino acid substitutions at L115P, I189K, E263A, S267P, L274P and L314P. The substitutions L274P, L298P, Y304H and L310P are expected to have a considerable effect on the pathogenicity and immune reaction of the virus as they are located nearby the antigenic site (AA265–273), as reported by Cane [52]. The amino acid analysis for RSV-A in the 24 AA duplicated region revealed that most samples (28 samples) contained three AA substitutions (L274P, L298P and Y304H) that were described also in strains from several countries in different years including Saudi Arabia (KU726088, KU726087, MH388036, MH388041, MK182714-16,18,09), Wuerzburg (JX912364), Germany (JX12364), Italy (KC858245, KC587959) and Japan (AB808774, AB808757). L310P substitution was also found in the duplicated region of isolates from Saudi Arabia (KU726088, KU726087, MH388036, MH388038, MH388041, MK182714-16,18,09), Japan (AB808774), India (AB808757) and Kenya (KF246641). Sequences from Saudi Arabia collected in 2014–2016 (GenBank accession numbers MH388031, MH388034, MH388038) only matched the L274P and L298P substitutions from our study. AA substitution at position E262K was seen in isolates from Lebanon (MG793382), Saudi Arabia (MH388033), South Africa (KC476746, HG711628-29), Germany (KF391432), Brazil (KU5812173) and Malaysia (JX256945, JX256955, JX256965). The amino acid substitution V303A was detected in 10 of our RSV group A samples and wasalso seen in isolates from Italy (KC858245), Croatia (KC587959), Saudi Arabia (MH388031, MH388036, MH388041, MK182714-16,18,09) and India (KF057865).

Some amino acid substitutions would cause a change in the potential O- and N-glycosylation profile from the prototype strain as from the ON1 Canadian RSV-A prototype virus. Some of these changes led to the loss of N-glycosylation sites such as S105P, T137K, T239A and N318T, which were found in three different samples, and T320A existed in 10 samples, while other changes caused gain in N-glycosylation sites such as Q127N, T198N and S315N. These amino acid changes were also found in many other strains from Kenya, Italy, India, Germany, Austria, Jordan, the USA, China, Egypt, Lebanon and New Zealand. These AA variations leading to the change in N- and O-glycosylation sites might enable the ON1 strain of RSV-A viruses to escape the immune system, as the polysaccharides play a masking role for the G protein, which prevents the antibodies from recognizing the virus [29].

For all RSV group B samples (Figure 3), phylogenetic analysis showed that they belonged to the BA strain, which was first detected in 1999 in Buenos Aires with 60 nucleotide duplication starting after residue 791 at the C-terminal in the second hypervariable region in the G gene [53]. All RSV group B samples belonged to BA9 strain. The BA9 genotype was also reported from other studies in Riyadh; the first study reported the detection of the subgroups BA4, BA7, BA10 and CB-Bin in samples collected in two winter seasons 2007–2008 and 2008–2009 [16]. The second Saudi study reported that all samples collected in 2014 belonged to BA9 [6]. The sequences of the BA9 strain from this study clustered with the sequences form Saudi Arabia, the Philippines, Thailand, Japan, China, Argentina, India, New Zealand and Brazil.

The deletion of CCAAAA nucleotides in all samples of RSV-B in this study caused the deletion of two PK amino acids, which was also reported in Saudi [6,16] and other countries in different isolation years, such as in Belgium with GenBank accession number AY751087, Saudi Arabia (JF714707, JF714708, KC719696), China (KT781360-82), the USA (KU950467/82), Jordan (KX655648), New Zealand (KX765906), the UK (KY249667), the Philippines (LC311362, LC311394), Japan (LC324679), Argentina (MG839547), Thailand (KC342333) and Australia (MH760729) [4,16,42,43,54,55]. Substitution at residue 254 T/I was found in 13 sample sequences from this study and was also found only in the Saudi samples in the 2016 study. The change of T/N at position 227 detected in 7 samples from this study was not found in other Saudi strains, causing the gain of a potential N-glycosylation site. The further mutations that were identified in the duplicated region of BA and ON1 genotypes indicate the gradual accumulation of mutations with time.

Anti-G broadly neutralizing monoclonal antibodies (bnmAbs) neutralize the virus and reduce the pathogenesis and the viral loads in both prophylactic and post-infection animal models. Its development as a vaccine had been held back due to heterogeneous N- and O-glycosylation in the highly-variable mucin-like regions and lack of information correlating a specific molecular structure with biological activity. This raises the importance of the molecular epidemiological data generated in this study and others that may help to provide more information about the mutation patterns of RSV, which could guide vaccine design and antiviral drug development.

We were not able to compare the full N- and O-glycosylation profiles between the Jeddah strain (N-glyco.: 4–8, O-glyco.: 52–86) and Riyadh strain (N-glyco.: 3–4, O-glyco.: 29–77) as the sequences reported from Riyadh were shorter and they were only focusing on the second hypervariable region. The clustering of Jeddah sequences with strains from different regions of the world indicates the possible introduction of a wide distribution of RSV isolates with visitors to the city from all over the world.

## 5. Conclusions

In this report, we have identified the full genome of RSV-A from Saudi Arabia for the first time and showed the genetic diversity of RSV in Jeddah at the time of the study compared to earlier studies in Saudi Arabia. The current study adds to the gap in knowledge about the genetic investigation of the virus in this geographically important part of the county. The international flux of travelers through Jeddah during hajj and umrah seasons as compared to Riyadh and Taif increases the chance of introducing new RSV strains and genotypes. Results from this study showed the predominance of the ON1 genotype of RSV-A and BA-9 genotype of RSV-B in Jeddah for the year of study as compared to the previous studies in Saudi Arabia that showed high prevalence of the NA1 genotype of RSV-A and other genotypes of RSV-B. Genetic characterization of the circulating respiratory infections including RSV is very crucial for the healthcare authorities to be able to take the needed preventive and treatment measures in combating these respiratory pathogens and for the scientific community to increase the background data to help in vaccine and antiviral development.

## Figures and Tables

**Table 1:** Demography of respiratory syncytial virus (RSV) positive samples.

		Frequency N (%)
Age	<1 year	60 (71.4)
	1–7 years	9 (10.7)
	>7 years	15 (17.9)
Gender	F	36 (42.9)
	M	48 (57.1)
Month	January 17	7 (8.3)
	March 17	2 (2.4)
	September 17	2 (2.4)
	October 17	2 (2.4)
	November 17	28 (33.3)
	December 17	43 (51.2)
Ward	ER	57 (67.9)
	ISO	3 (3.6)
	MIC	1 (1.2)
	PICU	23 (27.4)

N: number of infected cases; (%): within the brackets represents the percent of cases.

**Table 2:** The nucleotide variation between the RSV-A Jeddah strains and the ON1 prototype.

Sequence Name	Position Site	ON1 Nucleotide	Variant Nucleotide	Amino Acid Change	Codon Change	Protein Effect
**JN257694-Canada-2011**	910	U	C	Y to H	UAU to CAU	Substitution
	653	M	A	X to Q	CMA to CAA	Substitution
	908	U	C	V to A	GUC to GCC	Substitution
	704	C	T	T to I	ACC to ATC	Substitution
	958	A	G	T to A	ACA to GCA	Substitution
	617	C	A	P to Q	CCA to CAA	Substitution
	821	U	C	L to P	CUA to CCA	Substitution
	893	U	C	L to P	CUA to CCA	Substitution
	929	U	C	L to P	CUA to CCA	Substitution
	941	U	C	L to P	CUA to CCA	Substitution
	742	C	A	L to I	CUC to AUC	Substitution
	401	A	T	K to I	AAA to ATA	Substitution
	728	U	G	I to S	AUC to AGC	Substitution
	784	G	A	E to K	GAG to AAG	Substitution
	69	G	A		CUG to CUA	None
	369	G	A		GAG to GAA	None
	717	U	C		ACU to ACC	None
	840	U	C		UAU to UAC	None
	945	U	C		UCU to UCC	None

**Table 3:** The nucleotide variation between the RSV-B Jeddah strains and the BA prototype.

Sequence Name	Position Site	BA Nucleotide	Variant Nucleotide	Amino Acid Change	Codon Change	Protein Effect
**AY333364-Argentina-1999**	30	G	A		AAG to AAA	None
	177	C	T		CUC to CUT	None
	276	C	A		AUC to AUA	None
	283	U	C	Y to H	UAC to CAC	Substitution
	286	C	T	L to F	CUU to TUU	Substitution
	329	U	C	L to P	CUC to CCC	Substitution
	334	A	G	T to A	ACC to GCC	Substitution
	339	A	G		ACA to ACG	None
	349	U	C	Y to H	UAC to CAC	Substitution
	375	U	C		CCU to CCC	None
	422	G	C	R to T	AGA to ACA	Substitution
	427	AC	TCT	T to S	ACC to TCT	Substitution
	490	CCAAAA		PK to no Amino Acid	CCA, AAA to no codon	Deletion
	525	C	T		UUC to UUT	None
	591	U	C		AAU to AAC	None
	612	C	T		ACC to ACT	None
	614	U	C	I to T	AUA to ACA	Substitution
	636	C	T		CCC to CCT	None
	663	A	C		CCA to CCC	None
	668	A	C	K to T	AAA to ACA	Substitution
	671	U	C	L to P	CUA to CCA	Substitution
	683	U	C	L to P	CUG to CCG	Substitution
	695	C	A	T to N	ACC to AAC	Substitution
	702	C	T		AUC to AUT	None
	732	C	T		ACC to ACT	None
	754	U	C	S to P	UCA to CCA	Substitution
	776	C	T	T to I	ACA to ATA	Substitution
	824	C	T	T to I	ACU to ATU	Substitution
	827	U	C	V to A	GUG to GCG	Substitution
	831	C	T		CUC to CUT	None
	857	U	C	I to T	AUC to ACC	Substitution
	870	C	T		UCC to UCT	None
	874	C	T	H to Y	CAC to TAC	Substitution
	927	C	T		UCC to UCT	None
	948	C	T		UCC to UCT	None
	952	C	T			Truncation
	955	AAA	CTC	K to L	AAA to CTC	Substitution
	956	AA	CT	K to T	AAA to ACT	Substitution
	959	UC	AG	L to Q	CUC to CAG	Substitution
	959	UC	CA	L to P	CUC to CCA	Substitution
	961	UAG	GTC			Extension
	962	AG	CA			Extension
	964	U	A			
	965	C	A			
	965	C	T			
	966	AU	TG			
	968	A	C			
	968	A	G			
	969	U	C			
	970	G	T			
	971	C	A			
	971	C	T			
	972	U	A			
	972	U	G			
	973	U	G			
	974	A	T			
	975	G	A			
	975	G	T			
	976	U	A			
	978	A	T			
	979	UUC	AAA			
